# Water insecurity is associated with greater food insecurity and lower dietary diversity: panel data from sub-Saharan Africa during the COVID-19 pandemic

**DOI:** 10.1007/s12571-023-01412-1

**Published:** 2023-11-16

**Authors:** Joshua D. Miller, Sera L. Young, Elizabeth Bryan, Claudia Ringler

**Affiliations:** 1 Department of Nutrition, Gillings School of Global Public Health, University of North Carolina at Chapel Hill, Chapel Hill, NC, USA; 2 Department of Anthropology, Northwestern University, Evanston, IL, USA; 3 Institute for Policy Research, Northwestern University, Evanston, IL, USA; 4 International Food Policy Research Institute, Washington, DC, USA

**Keywords:** Agriculture, Dietary diversity, Food security, Nutrition, Water security

## Abstract

There is growing recognition that water insecurity – the inability to reliably access sufficient water for all household uses – is commonly experienced globally and has myriad adverse consequences for human well-being. The role of water insecurity in food insecurity and diet quality, however, has received minimal attention. Data are from panel surveys conducted during 2020–21 among adults involved in smallholder agriculture in Niger (n = 364, 3 rounds), Nigeria (n = 501, 5 rounds), Senegal (n = 501, 5 rounds), and Ghana (n = 543, 5 rounds). We hypothesized that household water insecurity (measured using the brief Household Water Insecurity Experiences Scale) would be associated with greater individual food insecurity (using 5 of the 8 Food Insecurity Experiences Scale items) and lower dietary diversity (using the Minimum Dietary Diversity Score for Women). At baseline, 37.1% of individuals were living in water-insecure households and of these, 90.6% had some experience of food insecurity. In multilevel mixed-effects regressions, individuals living in water-insecure households had 1.67 (95% CI: 1.47, 1.89) times higher odds of reporting any food insecurity experience and were estimated to consume 0.38-fewer food groups (95% CI: −0.50, −0.27) than those living in water-secure households. Experiences with suboptimal water access and use are associated with poor nutrition. The pathways by which water insecurity impacts nutrition should be identified. Global and national food and nutrition security policies could be strengthened by monitoring and developing strategies to address household water insecurity.

## 1 Introduction

Food and water are essential for human health and well-being, yet billions of individuals worldwide regularly experience problems accessing these critical resources in appropriate quantities and quality (Damania et al., 2020; Mekonnen & Hoekstra, 2016; Ringler & Paulo, 2020; Young et al., 2022). Experiences of food and water insecurity are projected to become more frequent, widespread, and acute due to climate change, infrastructural and environmental degradation, and greater demand from growing populations (Change, 2022; Hanjra & Qureshi, 2010; Srinivasan et al., 2012). The COVID-19 pandemic and associated mobility restrictions have caused supply chain disruptions and financial instability, imposing additional barriers to reliably acquiring food and water, both at household and national levels (Calder et al., 2021; FAO et al., 2021). These shocks have the potential to precipitate public health crises because unreliable access to sufficient and acceptable food and water causes psychological distress and increases the risk of both communicable and non-communicable diseases (Miller, Workman, et al., 2021; Rosinger & Young, 2020; Young, Bethancourt et al., 2021a; Young, Frongillo et al., 2021b; Young, Miller et al., 2021c). As such, there have been numerous calls to better understand the relationship between food and water insecurity, both to identify who is most affected and mitigate their deleterious effects (Miller, Workman, et al., 2021; Ringler & Paulo, 2020). Few studies, however, have quantitatively examined these factors, such that meaningful barriers to good nutrition and health may be regularly overlooked (Young, 2021).

There are many pathways by which water insecurity, a multi-dimensional construct that includes problems with water availability, access, use, and stability (Jepson et al., 2017), can undermine diets and nutritional well-being (Wutich & Brewis, 2014). The food production and income pathways have received the most attention to date: water insecurity can limit agricultural production, crop yields, and farm incomes, reducing the availability and affordability of nutrient-dense foods. Prior research has found that households that rely exclusively on rainfed agriculture have less diverse diets than their counterparts with access to irrigation technologies (Baye et al., 2021; Mekonnen et al., 2022; Passarelli et al., 2018). Importantly, though, water insecurity can also restrict the ability of individuals to implement best hygiene practices (e.g., washing hands) (Hannah et al., 2020), which in turn increases exposure to pathogens and pollutants that can decrease nutrient absorption (Young, Bethancourt et al., 2021a; Young, Frongillo et al., 2021b; Young, Miller et al., 2021c). Additionally, managing the burden of water insecurity presents opportunity costs that can undermine nutrition. For example, women and children in water-insecure households often have greater water-related time burdens (e.g., for fetching water) that restrict their engagement in education or income-generating activities (Miller, Workman, et al., 2021). This can reduce a household’s capacity to purchase (diverse) foods, thereby increasing risk of malnutrition. Finally, water insecurity can limit which foods are prepared. A cross-cultural qualitative study found that individuals substituted preferred dishes with meals using less diverse foods when water was scarce (Schuster et al., 2020).

Despite these many interlinkages between water and food, there is a dearth of research examining how water access and use influences nutritional well-being (Miller, Workman, et al., 2021; Young, Bethancourt et al., 2021a; Young, Frongillo et al., 2021b; Young, Miller et al., 2021c). A major reason that the role of water insecurity in food insecurity and diet quality is not well understood is that experiences with water access and use have traditionally not been measured. Instead, emphasis has been placed on physical water availability (e.g., m^3^ water/ capita) or drinking water infrastructure (e.g., availability of “improved” water sources) (Octavianti & Staddon, 2021). In contrast, the measurement of food insecurity is more mature; validated scales to comparably measure experiences of food access and use across diverse contexts have been available for several decades. The use of experiential measures of food insecurity has revealed inequalities that are masked by less comprehensive indicators – such as calories available per capita – and strong associations with many adverse health outcomes (Young, 2021). Although experiences of water insecurity are frequently reported globally and may undermine health and well-being to a similar magnitude as food insecurity, tools to holistically measure experiences with water access and use were only recently validated (Young et al., 2019; Young, Bethancourt et al., 2021a; Young, Frongillo et al., 2021b; Young, Miller et al., 2021c).

There is thus considerable need to empirically examine the relationships between water, food, and diets, especially in sub-Saharan Africa, where approximately half of the population directly relies on smallholder agriculture, which is particularly vulnerable to the water-related impacts of climate change (Ringler & Paulo, 2020). Understanding the nature of these relationships can help to identify at-risk individuals and inform the development of more targeted policies and programs that address proximal and distal determinants of poor nutrition (Young, 2021). We therefore used data from a panel study to assess, for the first time, the role of household water insecurity in food insecurity and diet quality among smallholder farmers. We hypothesized that greater household water insecurity would be associated with greater food insecurity and lower dietary diversity scores (a proxy of diet adequacy that is inversely associated with risk of nutrient deficiency).

## 2 Methods

### 2.1 Study settings and design

Data are from a subset of sites included in a panel study that sought to understand the differential impact of the COVID-19 pandemic and lockdown measures on health and well-being among adult (≥ 18 years) men and women engaged in agriculture. Three sites were excluded (Kenya, Uganda, Nepal) because fewer than three rounds of data were collected or a modified version of the water insecurity tool that has not been cross-culturally validated was used. Participants were eligible if they had participated in previous face-to-face surveys that were conducted prior to the pandemic and had access to a telephone (mobile or landline).

Surveys were implemented from July 2020 to July 2021. Sampling frames for the telephone surveys differed across sites to align with the original aims of their respective studies. In Ghana, the sample was drawn from two USAID-funded projects in the Upper East and Northern Regions: the “Innovation Laboratory for Small-Scale Irrigation” survey (n = 380) and the “Africa Rising” survey (n = 163). In Niger, participants were sampled from a study (“Local Economy Effects of Migration”) conducted in Maradi and Tillaberi (n = 237) and a study (“Social Network Analysis”) around Lake Chad in the Diffa Region (n = 280). Sites in Ghana and Niger were pooled for analysis because of contextual similarities. In Nigeria, participants were sampled from the “Agro-Processing Productivity Enhancement and Livelihood Improvement Support” survey, implemented by the Nigeria Ministry of Agriculture and supported by the World Bank. Five hundred individuals were sampled across two states (Kaduna and Cross River) located in Nigeria’s Feed the Future zone. These sites were analyzed separately given substantial differences in climate and water infrastructure. The sample in Senegal was drawn from a 2017 project (“Projet d’Appui aux Politiques Agricoles”), implemented in dry cereal producing areas by the Senegalese Ministry of Agriculture and Rural Equipment.

Five survey rounds were conducted in Ghana, Nigeria, and Senegal, and three in Niger because it was added later (Supplementary Fig. 1). Intervals between surveys ranged from 1 to 3 months. Households were randomly selected and one respondent from each was surveyed; women were purposively selected to maintain gender parity. Households were replaced due to attrition between rounds in the parent study, but for this analysis, only data from individuals who participated in the baseline survey are included.

Data collection tools and study protocols were approved by IFPRI’s Institutional Review Board. Informed consent was verbally obtained.

### 2.2 Data collection

Study staff administered 20- to 30-min-long structured questionnaires using the Computer-Assisted Telephone Interviewing (CATI) system; responses were recorded through the SurveyCTO platform. Interviews were conducted in local languages in Ghana and Nigeria, French and Hausa in Niger, and French and Wolof in Senegal. To ensure respondent privacy, interviewers confirmed that participants were in private spaces and checked for speakerphone use before asking sensitive questions. Participants were given phone credits (approximately $1 USD in value) after each survey round.

### 2.3 Exposure: household water insecurity

Household water insecurity was measured using the 4-item version of the Household Water Insecurity Experiences Scale (HWISE-4), which captures the prevalence of experiences with suboptimal water accessibility and use (Young, Bethancourt et al., 2021a; Young, Frongillo et al., 2021b; Young, Miller et al., 2021c). Participants were asked to report how frequently they worried about having insufficient water for household needs, changed plans because of problems with water, did not have enough water to drink, or were unable to wash hands because of problems with water in the prior two weeks. Responses [0 = never (0 times), 1 = rarely (1 time), 2 = sometimes (2–5 times), 3 = often (6–10 times)/always (> 10 times)] were summed together (range: 0–12), with higher scores indicating greater water insecurity; households with scores ≥ 4 were considered water insecure (Young, Bethancourt et al., 2021a; Young, Frongillo et al., 2021b; Young, Miller et al., 2021c). The scale had high internal consistency across sites and time points (Cronbach’s alpha: 0.83–0.95).

### 2.4 Outcomes: individual food insecurity and dietary diversity

To reduce respondent burden, individual food insecurity was assessed using a subset of items from the 8-item Food Insecurity Experiences Scale (FIES) that were expected to have the greatest variation (Ballard et al., 2013). These were: worrying about not having enough to eat, being unable to eat healthy or nutritious foods, skipping meals, eating less than desired, and going hungry in the prior two weeks. Affirmative responses were scored as 1 and summed (range: 0–5); higher scores indicate greater food insecurity. The scale had reasonable internal consistency across sites and time points (Cronbach’s alpha: 0.70–0.87).

Dietary diversity was measured using the Minimum Dietary Diversity Score for Women (MDD-W), an indicator designed for use among women of reproductive age but found to be suitable among men (Bromage et al., 2021). Individuals were asked whether they had consumed any foods from 10 food groups in the prior 24 h (range: 0–10). Three binary variables were generated to capture the consumption of animal-source foods (meat, eggs, or dairy; an important protein source in low- and middle-income countries), grains or pulses (typical household staples), and fruits or vegetables (critical sources of essential micronutrients) in the prior 24 h.

### 2.5 Covariates

Participants provided information about their gender (male/female), age (years), education (none, primary, secondary or beyond), and whether they were currently married (yes/no). Participants also reported number of household members, primary source of drinking water, whether they experienced income loss or changes to food access due to the pandemic, and if COVID-19-related mobility restrictions limited their ability to buy food or acquire water or firewood. A four-level categorical variable was developed to indicate whether individuals were involved in agriculture, owned livestock, both, or neither. A season variable (dry/wet) was generated based on date of interview and historical rainfall patterns.

### 2.6 Data analysis

Baseline respondent characteristics were described using univariable analysis. Water insecurity, dietary diversity, and food insecurity data were missing for 9.5%, 9.7%, and 9.9% of observations, respectively. Data were assumed to be missing at random. Multiple imputation with chained equations was used to generate values for missing instances of the primary exposure (water insecurity) and outcomes of interest. Predictive mean matching was used for continuous variables, logistic regression for binary variables, and multinomial logistic regression for categorical variables. To evaluate the impact of missing data, we performed all analyses using both the observed data (complete-case analysis) and by pooling across ten imputed datasets. Results from complete-case analysis are presented in the main text and results using imputed data in the supplementary materials; direction and magnitude of associations were similar across these datasets (Supplementary Tables 1 and 2).

Multilevel mixed-effects regression models were used to assess the association between water insecurity and both food insecurity and dietary diversity, accounting for repeated observations within individuals and clustering by site. For each outcome, water insecurity was treated as both as a continuous score and binary indicator. Analogous models were also developed with lagged water insecurity (i.e., water insecurity status from the prior survey round) as the main predictor. Given that the MDD-W was developed for use among women, we assessed whether gender modified the relationship between water insecurity and dietary diversity by including it as an interaction term. The likelihood ratio test suggested that inclusion of the interaction did not improve fit (*p* = 0.880); results are therefore not disaggregated by gender. Models were adjusted for putative confounders: gender, age, marital status, education level, household size, smallholder farmer status, season of interview, survey wave, water source, food insecurity score (for models of dietary diversity only), and COVID-19-related income loss, mobility restrictions, and changes to food access. Analyses were two-tailed tests (α = 0.05) and completed using Stata 17.0 (StataCorp LLC; College Station, TX).

## 3 Results

### 3.1 Study population

Cohort characteristics varied across study sites (Table 1). For instance, most respondents in Niger and Senegal were men (85.8% and 84.4%, respectively) whereas the proportion of men and women was nearly 1:1 elsewhere. Most participants were married, 87.3% had experienced a loss of income due to COVID-19 at baseline, and 60.0% used piped water as their primary drinking water source. Most participants (76.5%) raised crops and livestock; only 6.7% of participants reported not engaging directly in agriculture at the time of the survey.

At baseline, approximately one-third of individuals were from water-insecure households and the mean food insecurity score was 3.0 (sd: 1.9) (Table 1). Among participants in water-insecure households at baseline, most (90.6%) reported one or more food insecurity experiences. Dietary diversity was moderate across sites; on average, participants consumed 5 of 10 potential food groups.

Food insecurity was persistently high across visits in Ghana and Nigeria sites; it was less severe in Niger (Supplementary Fig. 1). Within sites, there was little temporal variation in food and water insecurity experiences, even though survey rounds occurred across rainy and dry seasons.

### 3.2 Food insecurity

**Fig. 1:**
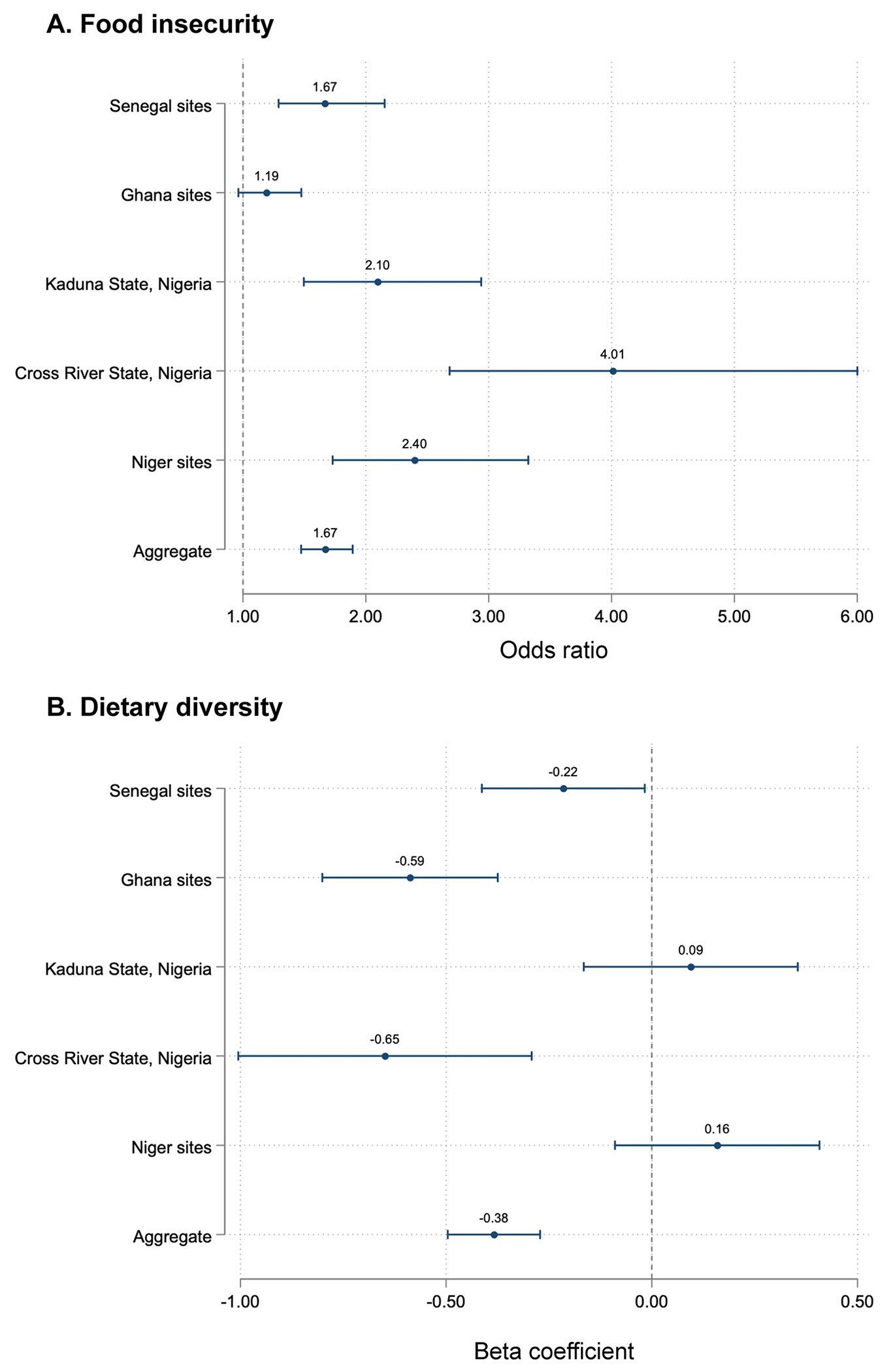
Associations between household water insecurity (HWISE-4 score ≥ 4) and **A** odds of reporting at least one experience of food insecurity in the prior two weeks, and **B** individual dietary diversity (MDDW Score, 0–10), in a panel study among adults engaged in agriculture in sub-Saharan Africa in 2020–2021, by study region and in aggregate. Point estimates and 95% confidence intervals estimated using multilevel mixed-effects ordered logistic and linear regressions, adjusting for putative confounders

As hypothesized, higher water insecurity scores were associated with higher odds of reporting more food insecurity experiences across most sites and in aggregate (Table 2, Fig. 1A). In a multilevel mixed-effects ordered logistic regression, individuals in water-insecure households had 1.67 (95% CI: 1.47, 1.89) times higher odds of reporting any food insecurity experience compared to their water-secure counterparts. In a multilevel mixed-effects linear regression, each point higher on the Household Water Insecurity Experiences Scale was associated with 1.07 times the odds of being food insecure (95% CI: 1.05, 1.09). Water insecurity experiences at one time point were not found to be predictive of food insecurity at the subsequent call (Supplementary Table 3).

### 3.3 Dietary diversity

**Fig. 2:**
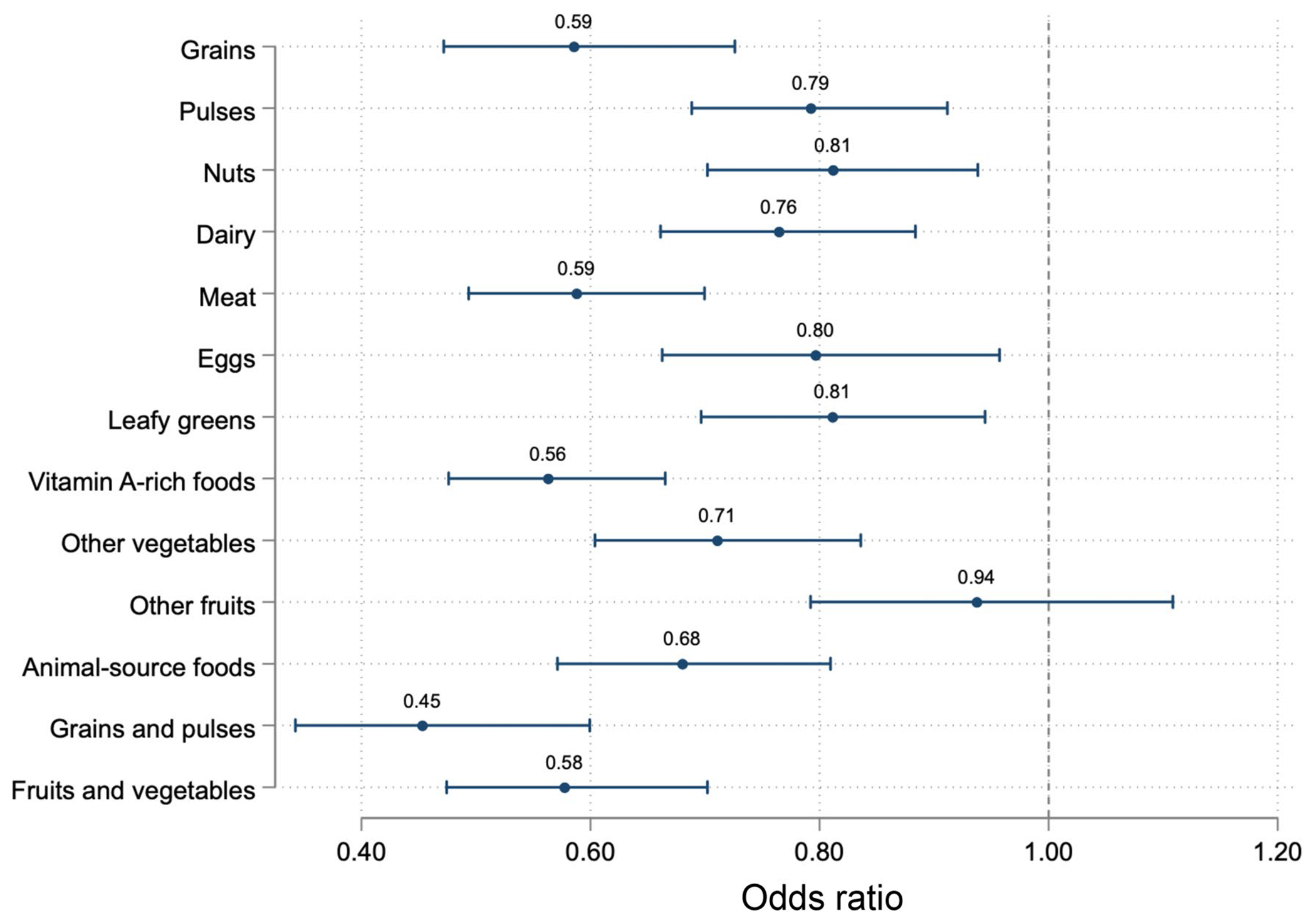
Associations between household water insecurity (HWISE-4 score ≥4) and 10 food groups included in the MDD-W and 3 food categories in a panel study among adults engaged in agriculture in sub-Saharan Africa in 2020–2021. Point estimates and 95% confidence intervals estimated using multilevel mixed-effects logistic regressions, adjusting for putative confounde

Higher water insecurity scores were associated with lower MDD-W scores (Table 2, Fig. 1B). Relative to individuals living in water-secure households, individuals in water-insecure households were estimated to consume 0.38-fewer food groups (95% CI: −0.50, −0.27). Individuals from water-insecure households had lower odds of consuming each type of food included in the MDD-W, except for fruits (Fig. 2, Supplementary Table 4). Individuals in water-insecure households had lower odds of consuming animal-source foods (OR: 0.68, 95% CI: 0.57, 0.81), grains or pulses (OR: 0.45, 95% CI: 0.34, 0.60), and fruits or vegetables (OR: 0.58, 95% CI: 0.47, 0.70) relative to their water-secure counterparts (Table 3). Similar trends were observed when water insecurity was modeled continuously.

Household water insecurity at one time point was negatively associated with dietary diversity at the subsequent interview. Individuals in water-insecure households were estimated to consume 0.11-fewer food groups at the next survey wave relative to those in water-secure households, although plausible magnitudes of association ranged from 0.24-fewer to 0.03-greater food groups (Supplementary Table 3). Greater water insecurity was also associated with lower odds of consuming grains or pulses (OR: 0.94, 95% CI: 0.90, 0.99) and fruits or vegetables (OR: 0.95, 95% CI: 0.92, 0.99) at the subsequent visit (Supplementary Table 5).

## 4 Discussion

In this first longitudinal assessment of food and water insecurity among smallholder farmers in sub-Saharan Africa, water insecurity was commonly experienced and found to be a risk factor for both food insecurity and lower dietary diversity. Individuals from water-insecure households were more likely to report experiences of food insecurity in the prior two weeks and less to likely to consume animal-source foods, grains or pulses, or fruits or vegetables in the prior 24 h relative to their water-secure counterparts. Water insecurity experiences reported at the prior point of contact were not associated with current food insecurity but were associated with lower dietary diversity.

Positive relationships between water and food insecurity have been observed in other populations (Boateng et al., 2020; Brewis et al., 2020; Miller, Frongillo et al., 2021a, Miller, Workman et al., 2021b; Young et al., 2019). The tools used to measure each resource insecurity differ across studies, such that strengths of association cannot be compared. For instance, the 23-country study used to develop the 12-item Household Water Insecurity Experiences Scale (from which the 4-item scale used herein was adapted) found that each point higher on the scale was associated with 0.38-points higher on the Household Food Insecurity Access Scale (Young et al., 2019). Consistent findings across studies, however, suggest that water and food insecurity often co-occur, and that water insecurity may increase the risk of food insecurity. Smallholder farmers who experience water insecurity may be unable to implement improved agricultural water management practices, such as irrigation, that enhance crop growth and yields (Mancosu et al., 2015). Additionally, prior work has found that some individuals report managing water stress by postponing plant cultivation (Venkataramanan et al., 2020). As such, water-insecure households engaged in agriculture may produce less food to consume or to sell at market and thereby have less food purchasing power, although these theoretical pathways need to be tested empirically (Ringler & Paulo, 2020).

This is the first study to examine the relationship between experiential water insecurity and dietary diversity, such that it is difficult to contextualize our findings. The observed magnitude of association (0.38-fewer food groups among individuals from water-insecure compared to water-secure households) is substantial and similar to that reported in studies examining the association between food insecurity and dietary diversity (Hashmi et al., 2021; Kang et al., 2019). Our findings are consistent with qualitative evidence that experiences with water insecurity limit a household’s capacity to cook preferred meals that incorporate ingredients from multiple food groups (Collins et al., 2019; Schuster et al., 2020). They also align with a previous study in India that found that suboptimal water access (one dimension of water insecurity) was associated with lower odds of meeting minimum dietary diversity among young children (Choudhary et al., 2021).

There are numerous potential mechanisms linking water insecurity to lower dietary diversity. For example, water is needed to make cereals (e.g., dried millet) and starchy tubers (e.g., cassava) palatable and digestible; experiences with water insecurity have been reported to negatively affect the ability to prepare these starches (Schuster et al., 2020). Interestingly, we found that water insecurity was associated with lower odds of consuming almost all food groups measured by the MDD-W, suggesting that individuals in water-insecure households may decrease consumption of most foods, not just starches. There was, however, a null association between water insecurity and odds of consuming fruits. A prior study in Bolivia found that individuals increased intake of water-rich fruits for hydration when they did not have access to safe drinking water (Rosinger & Tanner, 2015) whereas a study in Lebanon found that individuals could not wash fruits and vegetables when they experienced problems with water accessibility and quality and thus limited their intake (Korfali & Jurdi, 2008). It is possible that participants in this present study practiced both types of management strategies, leading to the null association. Future work should further explore which foods individuals prioritize when they experience water insecurity and whether such dietary decisions are context specific.

It is notable that water insecurity was predictive of subsequent dietary diversity but not future food insecurity. In the only other longitudinal study that measured experiential food and water insecurity concurrently, household water insecurity experienced by Kenyan women at 15 months postpartum did not predict food insecurity 3 months later, but water insecurity at 18 months postpartum predicted food insecurity at 21 months postpartum (Boateng et al., 2020). If food production is the most salient pathway between water and food insecurity among smallholder farmers, the observed lack of association may be due to the timing of surveys: most crops take more than 3 months to raise and harvest.

Taken together, these findings suggest that water insecurity can play a key role in nutritional outcomes. As such, interventions that aim to improve food security and dietary diversity should determine whether water insecurity is a contextually relevant barrier. Relatedly, policies and programs designed to reduce problems with water access and use should also measure impacts on nutritional well-being. Identifying unexpected benefits of improvements to water insecurity could garner more financial and public support for such activities.

The nature of the relationships between water insecurity, food insecurity, and nutrition needs to be better understood for policies to be appropriately designed and targeted. Specifically, additional research is needed to understand which factors – food production, income generation, women’s time poverty, food preparation, or enhanced hygiene and sanitation – are most sensitive to changes in water security. Findings from such work can be used to identify water-related factors that can be leveraged to improve overall dietary diversity as well as consumption of nutrient-dense foods. It would also be useful to assess the role of water insecurity in other nutrition-related outcomes, including duration of (exclusive) breastfeeding, child growth, micronutrient status, overweight and obesity, and chronic disease (Rosinger & Young, 2020). Future studies should also investigate whether the amount of food consumed (grams and kcal) varies between water-insecure and -secure individuals. Contextual factors including seasonal patterns of rainfall, soil health, crops grown, division of labor, and infrastructure should also be measured because they likely modify these mechanisms. For example, cross-sectional surveys found that access to irrigation was associated with greater agricultural incomes, which in turn were associated with higher dietary diversity scores among farmers in Ethiopia, but not Tanzania (Passarelli et al., 2018).

Strengths of the study include the first concurrent measurement of water insecurity, food insecurity, and dietary diversity in a population vulnerable to resource insecurities: smallholder farmers in four sub-Saharan Africa countries. The persistence of observed relationships across survey waves also provides greater validity to our findings. Limitations include the use of telephone surveys, which may have led to a biased sample; households without phones are likely to be poorer, older, and potentially more vulnerable to the negative impacts of COVID-19. Further, there was incomplete information about potential confounders, including household wealth (information about education level, household size, and income loss due to COVID-19 were used as proxy measures of wealth) and data about distance to each household’s primary drinking water source. Only a subset of items of the food and water insecurity scales were implemented, meaning some relevant experiences may have been excluded. Additionally, the presented findings are from a secondary analysis of existing data, such that the study may have been underpowered to detect small differences in the outcomes of interest. Future studies should be specifically designed and powered to evaluate the role of water insecurity in nutritional well-being. Finally, the plausibility of a causal relationship would be strengthened if data pertaining to some of the pathways by which these phenomena were associated (e.g., cooking techniques, food purchases, crops grown, time spent acquiring water) had been collected.

These limitations notwithstanding, a strong and persistent positive association was observed between water insecurity, food insecurity, and diet quality. These findings suggest that experiences of water insecurity are important, but often overlooked, upstream determinants of nutrition. Mitigation of water insecurity may reduce food insecurity, improve dietary diversity, and enhance efforts to address the global burden of malnutrition.

## Supplementary Material

Supplementary File

## Figures and Tables

**Table 1:** Baseline sociodemographic characteristics of participants in a four-country study examining the impacts of COVID-19 among adults engaged in agriculture in 2020–2021, by study site

	Total	Senegal	Ghana	Kaduna State, Nigeria	Cross River State, Nigeria	Niger

	N = 1,909	N = 501	N = 543	N = 252	N = 249	N = 364
Gender, n (%)						
Male	1,255 (65.9%)	423 (84.4%)	276 (50.8%)	124 (49.2%)	125 (50.2%)	307 (85.8%)
Female	648 (34.1%)	78 (15.6%)	267 (49.2%)	128 (50.8%)	124 (49.8%)	51 (14.2%)
Age, mean (sd)	42.9 (13.8)	52.4 (14.0)	39.9 (12.0)	37.7 (10.5)	39.1 (10.4)	40.7 (13.7)
Education, n (%)						
None	555 (29.2%)	115 (23.0%)	330 (61.0%)	17 (6.7%)	0 (0.0%)	93 (26.0%)
Primary	696 (36.7%)	330 (66.1%)	95 (17.6%)	35 (13.9%)	10 (4.0%)	226 (63.1%)
Secondary or beyond	647 (34.1%)	54 (10.8%)	116 (21.4%)	200 (79.4%)	238 (96.0%)	39 (10.9%)
Marital status, n (%)						
Single, divorced, or widowed	401 (21.1%)	64 (12.8%)	90 (16.6%)	31 (12.3%)	81 (32.5%)	135 (37.7%)
Married	1,502 (78.9%)	437 (87.2%)	453 (83.4%)	221 (87.7%)	168 (67.5%)	223 (62.3%)
Household size, mean (sd)	10.1 (6.6)	15.4 (8.6)	8.7 (4.0)	8.9 (5.5)	5.5 (2.5)	8.9 (4.8)
Experienced income loss due to COVID-19, n (%)						
No	243 (12.7%)	24 (4.8%)	47 (8.7%)	23 (9.1%)	28 (11.2%)	121 (33.4%)
Yes	1,664 (87.3%)	477 (95.2%)	496 (91.3%)	229 (90.9%)	221 (88.8%)	241 (66.6%)
Agricultural activity, n (%)						
No crops or livestock	127 (6.7%)	8 (1.6%)	17 (3.1%)	47 (18.7%)	52 (21.1%)	3 (0.8%)
Crops only	124 (6.5%)	25 (5.0%)	13 (2.4%)	19 (7.5%)	34 (13.8%)	33 (9.2%)
Livestock only	196 (10.3%)	30 (6.0%)	98 (18.0%)	34 (13.5%)	30 (12.2%)	4 (1.1%)
Crops and livestock	1,452 (76.5%)	437 (87.4%)	415 (76.4%)	152 (60.3%)	130 (52.8%)	318 (88.8%)
Primary drinking water source, n (%)						
Piped water	1,141 (60.0%)	280 (55.9%)	453 (83.4%)	91 (36.1%)	176 (70.7%)	141 (39.4%)
Dug well	566 (29.7%)	159 (31.7%)	60 (11.0%)	126 (50.0%)	22 (8.8%)	199 (55.6%)
Surface water (e.g., lake)	71 (3.7%)	2 (0.4%)	23 (4.2%)	1 (0.4%)	29 (11.6%)	16 (4.5%)
Water kiosk	68 (3.6%)	49 (9.8%)	2 (0.4%)	10 (4.0%)	5 (2.0%)	2 (0.6%)
Delivered by truck	22 (1.2%)	7 (1.4%)	0 (0.0%)	5 (2.0%)	10 (4.0%)	0 (0.0%)
Spring	21 (1.1%)	0 (0.0%)	3 (0.6%)	15 (6.0%)	3 (1.2%)	0 (0.0%)
Rainwater	13 (0.7%)	3 (0.6%)	2 (0.4%)	4 (1.6%)	4 (1.6%)	0 (0.0%)
Purchase at store	1 (0.1%)	1 (0.2%)	0 (0.0%)	0 (0.0%)	0 (0.0%)	0 (0.0%)
HWISE-4 score (0–12), mean (sd)	2.9 (3.5)	2.9 (4.0)	3.5 (3.3)	1.7 (2.4)	2.4 (3.0)	3.0 (3.8)
Water insecure (HWISE-4 score ≥ 4), n (%)						
No	1,186 (62.9%)	326 (65.1%)	283 (52.6%)	179 (74.3%)	169 (68.1%)	229 (64.1%)
Yes	699 (37.1%)	175 (34.9%)	255 (47.4%)	62 (25.7%)	79 (31.9%)	128 (35.9%)
Food insecurity score (0–5), mean (sd)	3.0 (1.9)	2.8 (1.8)	3.4 (1.8)	3.5 (1.8)	3.7 (1.7)	1.9 (1.8)
Water-insecure households that experienced any food insecurity, n (%)						
No	65 (9.4%)	20 (11.4%)	12 (4.7%)	9 (14.5%)	0 (0.0%)	24 (18.8%)
Yes	629 (90.6%)	155 (88.6%)	241 (95.3%)	53 (85.5%)	76 (100.0%)	104 (81.3%)
Dietary diversity score (0–10), mean (sd)	5.3 (2.2)	4.6 (1.8)	6.4 (2.5)	5.2 (2.2)	5.9 (2.4)	4.3 (1.4)
Consumed animal-source foods in prior 24 h, n (%)						
No	594 (31.2%)	119 (23.8%)	128 (23.6%)	100 (39.7%)	35 (14.1%)	212 (59.2%)
Yes	1,309 (68.8%)	382 (76.2%)	415 (76.4%)	152 (60.3%)	214 (85.9%)	146 (40.8%)
Consumed grains or pulses in prior 24 h, n (%)						
No	134 (7.0%)	25 (5.0%)	70 (12.9%)	20 (7.9%)	19 (7.6%)	0 (0.0%)
Yes	1,769 (93.0%)	476 (95.0%)	473 (87.1%)	232 (92.1%)	230 (92.4%)	358 (100.0%)
Consumed fruits or vegetables in prior 24 h, n (%)						
No	216 (11.4%)	77 (15.4%)	22 (4.1%)	36 (14.3%)	42 (16.9%)	39 (10.9%)
Yes	1,687 (88.6%)	424 (84.6%)	521 (95.9%)	216 (85.7%)	207 (83.1%)	319 (89.1%)

**Table 2:** Multilevel mixed-effects ordered logistic and linear regressions of individual food insecurity and dietary diversity based on data from a four-country panel study among adults engaged in agriculture in sub-Saharan Africa in 2020–2021

	Food insecurity (0–5)				Dietary diversity (0–10)			
	Crude		Adjusted^a^		Crude		Adjusted^b^	
	OR	95% CI	OR	95% CI	B	95% CI	B	95% CI
Water insecurity score (0–12)	1.07	1.06, 1.09	1.07	1.05, 1.09	−0.08	−0.10, −0.07	−0.07	−0.09, −0.05
Water insecure (HWISE-4 score ≥ 4)	1.60	1.43, 1.80	1.67	1.47, 1.89	−0.45	−0.54, −0.35	−0.38	−0.50, −0.27
Observations	8012		6131		8026		6092	

a Adjusted for respondent gender, age, marital status, education level, household size, smallholder farmer status, season of interview, survey wave, water source (piped or not), and COVID-19-related income loss, mobility restrictions, and changes to food access

b Adjusted for respondent gender, age, marital status, education level, household size, smallholder farmer status, season of interview, survey wave, water source (piped or not), and COVID-19-related income loss, mobility restrictions, changes to food access, and food insecurity score

**Table 3:** Multilevel mixed-effects logistic regressions of 24-h dietary intake based from a four-country panel study among adults engaged in agriculture in sub-Saharan Africa in 2020–2021

	Animal-source foods				Grains and pulses				Fruits or vegetables			

	Crude		Adjusted^a^		Crude		Adjusted^a^		Crude		Adjusted^a^	
	OR	95% CI	OR	95% CI	OR	95% CI	OR	95% CI	OR	95% CI	OR	95% CI
Water insecurity score (0–12)	0.93	0.91, 0.95	0.95	0.92, 0.97	0.87	0.84, 0.90	0.88	0.85, 0.92	0.90	0.88, 0.92	0.91	0.90, 0.94
Water insecure (HWISE-4 score ≥ 4)	0.59	0.51, 0.69	0.68	0.57, 0.81	0.44	0.34, 0.55	0.45	0.34, 0.60	0.52	0.44, 0.62	0.58	0.47, 0.70
Observations	8064		6122		8065		6125		8040		6102	

a Adjusted for respondent gender, age, marital status, education level, household size, smallholder farmer status, season of interview, survey wave, water source (piped or not), and COVID-19-related income loss, mobility restrictions, changes to food access, and food insecurity score

## Data Availability

The data that support the findings of this study are openly available at https://dataverse.harvard.edu/dataverse/IFPRI.
