# Supplementary material for: Water insecurity is associated with greater food insecurity and lower dietary diversity: panel data from sub-Saharan Africa during the COVID-19 pandemic
Source: Food Secur. Author manuscript; Available in PMC 2025 Feb 1. (PMC11784942; doi:10.1007/s12571-023-01412-1)
Supplement: Supplementary File [file NIHMS2009604-supplement-Supplementary_File.docx]

**Water insecurity is associated with greater food insecurity and lower dietary diversity: panel data from sub-Saharan Africa during the COVID-19 pandemic**

Joshua D. Miller, Sera L. Young, Elizabeth Bryan, Claudia Ringler

**Journal:** Food Security

**Corresponding author:**

Joshua D. Miller

Carolina Population Center

123 West Franklin St., Suite 210

Chapel Hill, NC 27516

United States

+1 (919) 962-5907

[josh.miller@unc.edu](mailto:josh.miller@unc.edu)


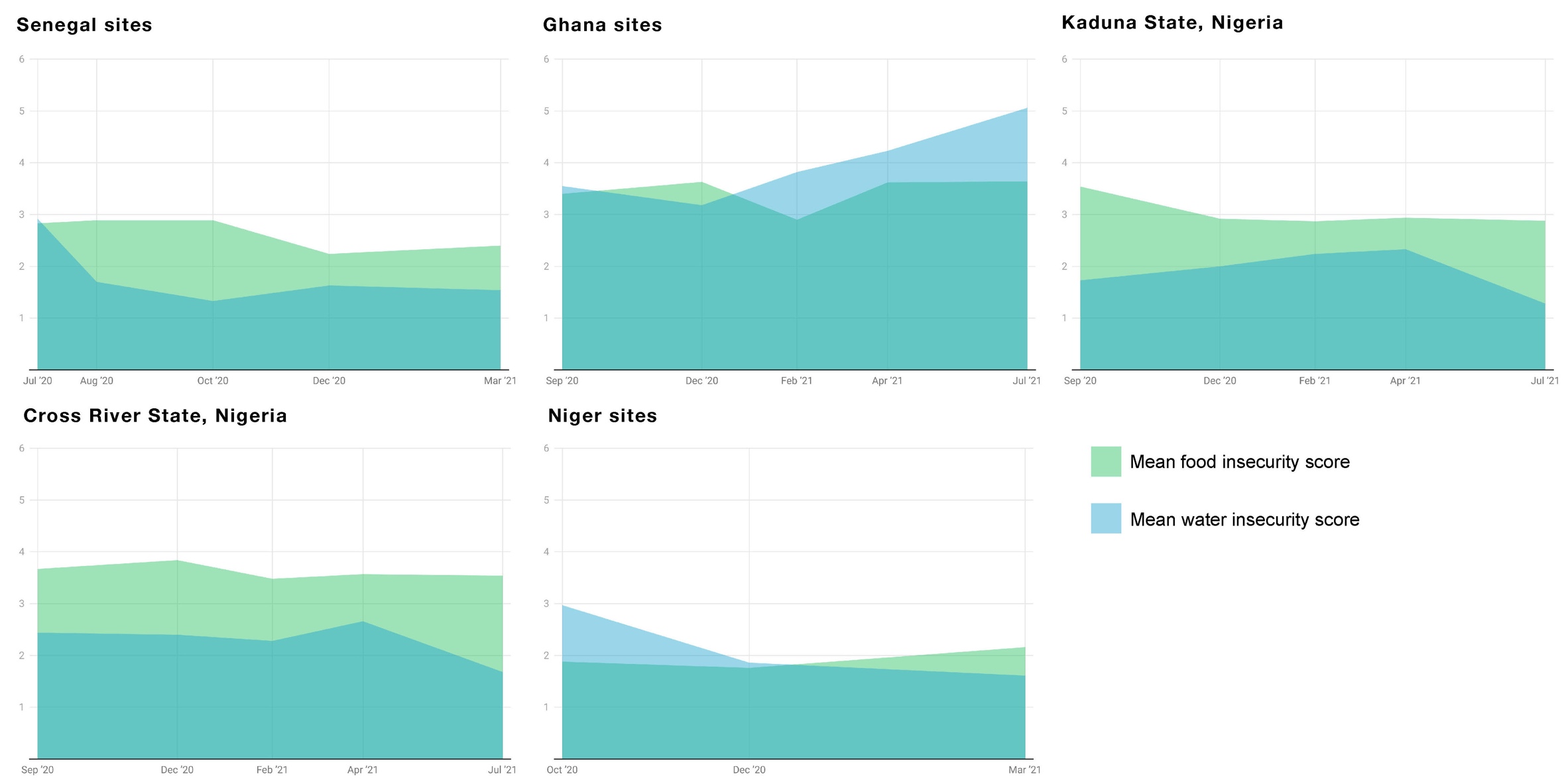


**Supplementary Fig. 1** Mean individual food insecurity and household water insecurity scores among adults involved in agriculture, by study site and sampling period

**Supplementary Table 1** Multilevel mixed-effects ordered logistic and linear regressions of individual food insecurity and dietary diversity based on ten imputed datasets, drawing on data from a four-country panel study among adults in sub-Saharan Africa engaged in agriculture

|  | **Food insecurity (0-5)** | | | | | **Dietary diversity (0-10)** | | | | |
| --- | --- | --- | --- | --- | --- | --- | --- | --- | --- | --- |
|  | Crude | | Adjusted^†^ | | | Crude | | Adjusted^‡^ | | |
|  | OR | 95% CI | | OR | 95% CI | B | 95% CI | | B | 95% CI |
| Water insecurity score (0-12) | 1.04 | 1.02, 1.06 | | 1.09 | 1.06, 1.11 | -0.08 | -0.09, -0.07 | | -0.08 | -0.10, -0.06 |
| Water insecure (HWISE-4 score≥4) | 1.36 | 1.22, 1.52 | | 1.75 | 1.48, 2.07 | -0.43 | -0.53, -0.34 | | -0.42 | -0.60, -0.25 |

^†^ Adjusted for respondent gender, age, marital status, education level, household size, smallholder farmer status, season of interview, survey wave, water source (piped or not), and COVID-19-related income loss, mobility restrictions, and changes to food access.

^‡^ Adjusted for respondent gender, age, marital status, education level, household size, smallholder farmer status, season of interview, survey wave, water source (piped or not), and COVID-19-related income loss, mobility restrictions, changes to food access, and food insecurity score.

**Supplementary Table 2** Multilevel mixed-effects logistic regressions of 24-hour dietary intake based on ten imputed datasets, drawing on data from a four-country panel study among adults in sub-Saharan Africa engaged in agriculture

|  | **Animal-source foods** | | | | **Grains and pulses** | | | | **Fruits or vegetables** | | | |
| --- | --- | --- | --- | --- | --- | --- | --- | --- | --- | --- | --- | --- |
|  | Crude | | Adjusted^†^ | | Crude | | Adjusted^†^ | | Crude | | Adjusted^†^ | |
|  | OR | 95% CI | OR | 95% CI | OR | 95% CI | OR | 95% CI | OR | 95% CI | OR | 95% CI |
| Water insecurity score (0-12) | 0.94 | 0.92, 0.96 | 0.95 | 0.92, 0.98 | 0.87 | 0.85, 0.90 | 0.86 | 0.81, 0.91 | 0.91 | 0.89, 0.93 | 0.95 | 0.92, 0.99 |
| Water insecure (HWISE-4 score≥4) | 0.64 | 0.55, 0.73 | 0.69 | 0.56, 0.85 | 0.44 | 0.35, 0.56 | 0.41 | 0.28, 0.60 | 0.54 | 0.46, 0.64 | 0.75 | 0.56, 1.02 |

^†^ Adjusted for respondent gender, age, marital status, education level, household size, smallholder farmer status, season of interview, survey wave, water source (piped or not), and COVID-19-related income loss, mobility restrictions, changes to food access, and food insecurity score.

**Supplementary Table 3** Multilevel mixed-effects ordered logistic and linear regressions of individual food insecurity and dietary diversity among adults in sub-Saharan Africa sampled in four countries, with lagged measures of water insecurity

|  | **Food insecurity (0-5)** | | | | **Dietary diversity (0-10)** | | | |
| --- | --- | --- | --- | --- | --- | --- | --- | --- |
|  | Crude | | Adjusted^†^ | | Crude | | Adjusted^‡^ | |
|  | OR | 95% CI | OR | 95% CI | B | 95% CI | B | 95% CI |
| Water insecurity score at previous survey (0-12) | 1.00 | 0.98, 1.02 | 0.99 | 0.97, 1.02 | -0.03 | -0.05, -0.02 | -0.03 | -0.05, -0.01 |
| Water insecure at previous survey (HWISE-4 score≥4) | 1.07 | 0.94, 1.23 | 1.01 | 0.86, 1.17 | -0.18 | -0.29, -0.07 | -0.11 | -0.24, 0.03 |
| Observations | 5642 |  | 5640 |  | 5640 |  | 4207 |  |

^†^ Adjusted for respondent gender, age, marital status, education level, household size, smallholder farmer status, season of interview, survey wave, water source (piped or not), and COVID-19-related income loss, mobility restrictions, and changes to food access.

^‡^ Adjusted for respondent gender, age, marital status, education level, household size, smallholder farmer status, season of interview, survey wave, water source (piped or not), and COVID-19-related income loss, mobility restrictions, changes to food access, and food insecurity score.

**Supplementary Table 4** Multilevel mixed-effects logistic regressions of 24-hour dietary intake based on data from a four-country panel study among adults in sub-Saharan Africa engaged in agriculture

|  | **Grains** | | | | **Pulses** | | | | **Nuts** | | | |
| --- | --- | --- | --- | --- | --- | --- | --- | --- | --- | --- | --- | --- |
|  | Crude | | Adjusted^†^ | | Crude | | Adjusted^†^ | | Crude | | Adjusted^†^ | |
|  | OR | 95% CI | OR | 95% CI | OR | 95% CI | OR | 95% CI | OR | 95% CI | OR | 95% CI |
| Water insecurity score (0-12) | 0.91 | 0.88, 0.93 | 0.92 | 0.89, 0.94 | 0.95 | 0.93, 0.97 | 0.95 | 0.93, 0.97 | 0.94 | 0.92, 0.96 | 0.96 | 0.94, 0.98 |
| Water insecure (HWISE-4 score≥4) | 0.59 | 0.49, 0.71 | 0.59 | 0.47, 0.73 | 0.81 | 0.72, 0.92 | 0.79 | 0.69, 0.91 | 0.72 | 0.63, 0.82 | 0.81 | 0.70, 0.94 |
| Observations | 8072 |  | 6130 |  | 8066 |  | 6125 |  | 8065 |  | 6124 |  |
|  | **Dairy** | | | | **Meat** | | | | **Eggs** | | | |
|  | Crude | | Adjusted^†^ | | Crude | | Adjusted^†^ | | Crude | | Adjusted^†^ | |
|  | OR | 95% CI | OR | 95% CI | OR | 95% CI | OR | 95% CI | OR | 95% CI | OR | 95% CI |
| Water insecurity score (0-12) | 0.95 | 0.93, 0.97 | 0.96 | 0.94, 0.98 | 0.90 | 0.88, 0.92 | 0.93 | 0.90, 0.95 | 0.95 | 0.93, 0.98 | 0.96 | 0.93, 0.99 |
| Water insecure (HWISE-4 score≥4) | 0.72 | 0.63, 0.81 | 0.76 | 0.66, 0.88 | 0.48 | 0.42, 0.56 | 0.59 | 0.49, 0.70 | 0.79 | 0.67, 0.93 | 0.80 | 0.66, 0.96 |
| Observations | 8069 |  | 6126 |  | 8072 |  | 6129 |  | 8066 |  | 6124 |  |
|  | **Leafy greens** | | | | **Vitamin A-rich foods** | | | | **Other vegetables** | | | |
|  | Crude | | Adjusted^†^ | | Crude | | Adjusted^†^ |  | Crude | | Adjusted^†^ |  |
|  | OR | 95% CI | OR | 95% CI | OR | 95% CI | OR | 95% CI | OR | 95% CI | OR | 95% CI |
| Water insecurity score (0-12) | 0.94 | 0.92, 0.96 | 0.95 | 0.93, 0.97 | 0.90 | 0.89, 0.92 | 0.91 | 0.89, 0.93 | 0.93 | 0.91, 0.95 | 0.94 | 0.91, 0.96 |
| Water insecure (HWISE-4 score≥4) | 0.73 | 0.64, 0.84 | 0.81 | 0.70, 0.94 | 0.54 | 0.47, 0.62 | 0.56 | 0.48, 0.67 | 0.69 | 0.60, 0.80 | 0.71 | 0.60, 0.84 |
| Observations | 8071 |  | 6128 |  | 8058 |  | 6117 |  | 8056 |  | 6117 |  |
|  | **Other fruits** | | | |  |  |  |  |  |  |  |  |
|  | Crude | | Adjusted^†^ | |  |  |  |  |  |  |  |  |
|  | OR | 95% CI | OR | 95% CI |  |  |  |  |  |  |  |  |
| Water insecurity score (0-12) | 0.98 | 0.96, 1.01 | 0.99 | 0.96, 1.01 |  |  |  |  |  |  |  |  |
| Water insecure (HWISE-4 score≥4) | 0.90 | 0.78, 1.04 | 0.94 | 0.79, 1.11 |  |  |  |  |  |  |  |  |
| Observations | 8053 |  | 6113 |  |  |  |  |  |  |  |  |  |

^†^ Adjusted for respondent gender, age, marital status, education level, household size, smallholder farmer status, season of interview, survey wave, water source (piped or not), and COVID-19-related income loss, mobility restrictions, changes to food access, and food insecurity score.

**Supplementary Table 5** Multilevel mixed-effects logistic regressions of 24-hour dietary intake among adults in sub-Saharan Africa sampled in four countries, with lagged measures of water insecurity

|  | **Animal-source foods** | | | | **Grains and pulses** | | | | **Fruits and vegetables** | | | |
| --- | --- | --- | --- | --- | --- | --- | --- | --- | --- | --- | --- | --- |
|  | Crude | | Adjusted^†^ | | Crude | | Adjusted^†^ | | Crude | | Adjusted^†^ | |
|  | OR | 95% CI | OR | 95% CI | OR | 95% CI | OR | 95% CI | OR | 95% CI | OR | 95% CI |
| Water insecurity score at previous survey (0-12) | 0.97 | 0.94, 0.99 | 0.98 | 0.95, 1.01 | 0.92 | 0.88, 0.96 | 0.94 | 0.90, 0.99 | 0.94 | 0.91, 0.97 | 0.95 | 0.92, 0.99 |
| Water insecure at previous survey (HWISE-4 score≥4) | 0.76 | 0.63, 0.92 | 0.86 | 0.68, 1.08 | 0.63 | 0.46, 0.86 | 0.74 | 0.51, 1.06 | 0.71 | 0.57, 0.89 | 0.81 | 0.64, 1.04 |
| Observations | 5675 |  | 4236 |  | 5677 |  | 4239 |  | 5653 |  | 4217 |  |

^†^ Adjusted for respondent gender, age, marital status, education level, household size, smallholder farmer status, season of interview, survey wave, water source (piped or not), and COVID-19-related income loss, mobility restrictions, changes to food access, and food insecurity score.
